# α‐Amylase expressed in human small intestinal epithelial cells is essential for cell proliferation and differentiation

**DOI:** 10.1002/jcb.29357

**Published:** 2019-09-03

**Authors:** Kimie Date, Tomomi Yamazaki, Yoko Toyoda, Kumi Hoshi, Haruko Ogawa

**Affiliations:** ^1^ Institute for Human Life Innovation Ochanomizu University, Ohtsuka, Bunkyo‐ku Tokyo Japan; ^2^ National Institute of Health and Nutrition National Institutes of Biomedical Innovation, Health and Nutrition, Toyama, Shinjuku‐ku Tokyo Japan; ^3^ Graduate School of Humanities and Sciences Ochanomizu University, Ohtsuka, Bunkyo‐ku Tokyo Japan

**Keywords:** caco‐2 cells, differentiation, expression, proliferation, small intestine, α‐amylase

## Abstract

α‐Amylase, which plays an essential role in starch degradation, is expressed mainly in the pancreas and salivary glands. Human α‐amylase is also detected in other tissues, but it is unclear whether the α‐amylase is endogenously expressed in each tissue or mixed exogenously with one expressed by the pancreas or salivary glands. Furthermore, the biological significance of these α‐amylases detected in tissues other than the pancreas and salivary glands has not been elucidated. We discovered that human α‐amylase is expressed in intestinal epithelial cells and analyzed the effects of suppressing α‐amylase expression. α‐Amylase was found to be expressed at the second‐highest messenger RNA level in the duodenum in human normal tissues after the pancreas. α‐Amylase was detected in the cell extract of Caco‐2 intestinal epithelial cells but not secreted into the culture medium. The amount of α‐amylase expressed increased depending on the length of the culture of Caco‐2 cells, suggesting that α‐amylase is expressed in small intestine epithelial cells rather than the colon because the cells differentiate spontaneously upon reaching confluence in culture to exhibit the characteristics of small intestinal epithelial cells rather than colon cells. The α‐amylase expressed in Caco‐2 cells had enzymatic activity and was identified as *AMY2B*, one of the two isoforms of pancreatic α‐amylase. The suppression of α‐amylase expression by small interfering RNA inhibited cell differentiation and proliferation. These results demonstrate for the first time that α‐amylase is expressed in human intestinal epithelial cells and affects cell proliferation and differentiation. This α‐amylase may induce the proliferation and differentiation of small intestine epithelial cells, supporting a rapid turnover of cells to maintain a healthy intestinal lumen.

**Abbreviations**
CNP: N3‐G5‐β‐2chloro4nitro phenol
DPP‐4: dipeptidylpeptidase‐4
GAPDH: glyceraldehyde‐3‐phosphate dehydrogenase
HSA: human salivary α‐amylase
HPA: human pancreatic α‐amylase
MTC: multiple tissue cDNA
MTT: thiazolyl blue tetrazolium bromide
SI: sucrase‐isomaltase
TBS: 10 mM Tris‐HCl buffered saline, pH 7.5

## 1 INTRODUCTION

α‐Amylase (EC 3.2.1.1) is a digestive enzyme that catalyzes the hydrolysis of internal α‐1,4‐glycosidic bonds of starch into smaller maltooligosaccharides. Pancreatic α‐amylase is essential for the acquisition of energy from dietary starch. On reaching the duodenum, the α‐amylase digests starch in the diet to small saccharides such as maltose for uptake in the small intestine. For a long time, it was thought that pancreatic α‐amylase was important only in starch digestion in the small intestine. However, we previously discovered that pancreatic α‐amylase binds with *N*‐linked oligosaccharides of glycoproteins.^1^ The binding activity regulates the activities of glycoproteins related to the blood glucose level, such as sucrase‐isomaltase (SI) and sodium/glucose cotransporter 1, on the small intestinal brush‐border membrane.^2^ In a study using differentiated Caco‐2 cells, we showed that the regulation is exerted by binding to *N*‐linked oligosaccharides on the small intestinal brush‐border membrane and is abolished by entering intestinal epithelial cells, resulting in blood glucose homeostasis.^3^ Caco‐2 cells were originally a human colon epithelial cell line but have developed characteristics of small intestine epithelium, such as expression of brush‐border membrane proteins on the apical surface when cultured for about 2 weeks after confluence.^4^ Differentiated Caco‐2 cells are one of the best models of intestinal epithelial cells and thus are widely used to reveal small intestinal functions such as absorption of various nutrients.^5^

Mammalian α‐amylase is mainly synthesized by the pancreas and salivary glands. α‐Amylases are detected in not only in pancreas and salivary glands but also in urine and blood.^6^ α‐Amylase is detected in rat liver, intestine, stomach, testis, and skeletal muscle as messenger RNA (mRNA) expression and starch degrading activity.^7^ Human α‐amylases have been detected in normal liver and thyroid tissues by reverse transcription polymerase chain reaction (RT‐PCR), assays of amylase activity using a starch method, histochemical analyses, and electrophoretic analysis.^8^, ^9^, ^10^ There are three α‐amylase genes, *AMY1*, *AMY2A*, and *AMY2B*, in a cluster on chromosome 1 P21. *AMY1* is the salivary α‐amylase gene and *AMY2A* and *AMY2B* are pancreatic α‐amylase genes.^11^ The gene sequence homologies of *AMY1A* and *AMY2A*, *AMY2B* are 93.2% and 93.6%, respectively, and that of *AMY2A* and *AMY2B* is 94.0%.^12^ The human α‐amylases detected in liver and thyroid tissues are *AMY2B* and *AMY1*, respectively.^8^, ^10^ Recently, it was reported that active α‐amylase is expressed in the human brain and that its isotypes are *AMY1A* and *AMY2A*.^13^ One report showed that α‐amylase was detected in 22 different tissues by electrophoresis and wheat‐gram inhibition, which is one of the α‐amylase activity assays.^14^ However, the biological significances of the expression of α‐amylases in these tissues except the salivary gland and pancreas have not been elucidated. The α‐amylase detected in these tissues, especially the intestine, is not distinguished from endogenous α‐amylases expressed in each tissue and contaminated by exogenous α‐amylases produced by the pancreas or salivary glands. Because pancreatic α‐amylase binds to the intestinal brush‐border membrane and digests dietary starch in the small intestine.^2^, ^3^

In the present study, we discovered that human α‐amylase is expressed in intestinal epithelial cells, and ascertained that the α‐amylase is encoded by the *AMY2B* gene. Additionally, to determine the biological significance of α‐amylase expression in small intestinal epithelial cells, we analyzed the effects of suppressing α‐amylase expression.

## 2 MATERIALS AND METHODS

### 2.1 Reagents and antibodies

Caco‐2, which is a human colon cell line, was purchased from RIKEN Cell Bank (Tsukuba, Japan). Minimum essential medium eagle (MEM) and thiazolyl blue tetrazolium bromide were purchased from Sigma‐Aldrich, Co (St Louis, MO). Phosphate buffered saline (PBS) pH 7.2 (×10), MEM non‐essential amino acids (NEAA) (×100), fetal bovine serum (FBS), OPTI‐MEM I reduced serum media (×1), 0.05% trypsin‐ethylenediaminetetraacetic acid (×1) phenol red, TRIzol reagent, PowerSYBER Green PCR Master Mix, and 0.4% trypan blue stain was purchased from Life Technologies (Carlsbad, CA, Warrington, UK, or Grand Island, NY). siLentFect lipid was purchased from Bio‐Rad Laboratories, Inc. (Hercules, CA). dNTPs mixture and ReverTra Ace were purchased from Toyobo Co, Ltd (Osaka, Japan). Random primers and Sypro Ruby were purchased from Invitrogen (Carlsbad, CA). CELLBANKER1 was purchased from ZENOAQ (Fukushima, Japan). 4′,6‐Diamidino‐2‐phenylindole (DAPI) was purchased from Roche Diagnostics GmbH, (Mannheim, Germany). Fluoromount‐G was purchased from Southern Biotech (Birmingham, AL). Pierce BCA protein assay kit was purchased from Thermo Fisher Scientific (Rockford, IL). An α‐amylase assay kit was purchased from Kikkoman Corp (Chiba, Japan). Amylase small interfering RNA (siRNA) (h) (sc‐29675) and control siRNA‐A (sc‐37007) were purchased from Santa Cruz Biotechnology, Inc (Dallas, TX). Pig pancreas α‐amylase was purchased from Elastin Products Company, Inc. (Owensville, MO). Human Multiple Tissue cDNA (MTC) Panels I, II, and a Human Digestive System MTC Panel were purchased from Clontech Laboratories, Inc (Mountain View, CA). Thiazolyl blue tetrazolium bromide (MTT) was purchased from Sigma‐Aldrich Co.

Rabbit anti‐α‐amylase immunoglobulin Gs (IgGs) to human pancreatic α‐amylase (anti‐HPA IgGs, K50894R) were purchased from Meridian Life Science, Inc (Memphis, TN). Horseradish peroxidase (HRP) goat anti‐rabbit IgGs as a secondary antibody were purchased from Kirkegaard & Perry Laboratories, Inc (Gaithersburg, MD). AlexaFluor 488 goat anti‐rabbit IgG (H+L) as a secondary antibody was purchased from Life Technologies (Invitrogen, Eugene, Oregon). Chemical reagents were purchased from Fujifilm Wako Pure Chemicals Corporation (Osaka, Japan) or Nacalai Tesque Inc (Kyoto, Japan).

### 2.2 Cell culture

Caco‐2 cells were cultured in MEM containing 20% heat‐inactivated (56°C, 30 minutes) FBS and 0.1 mM NEAA at 37°C under a humidified atmosphere of 95% air and 5% CO_2_. Caco‐2 cells were seeded at 0.5‐2.0 × 10^5^ cells/cm^2^. The culture medium was renewed every 2 or 3 days.

### 2.3 Sample preparation for Western blot analysis and starch degrading activity

Caco‐2 cells were seeded at 5 × 10^4^ cells/cm^2^, and the cells were cultured for 0 to 21 days. Culture medium was collected and the cells were washed twice with PBS. The cells were harvested by scraping into cold PBS and transferred to tubes. The cells were homogenized in 1 vol of 10 mM Tris‐HCl buffered saline, pH 7.5 (TBS) containing 2 mM phenylmethylsulfonyl fluoride (PMSF) using a glass‐Teflon homogenizer (HK‐1; As one Corp), 30 strokes at 1000 rpm on ice, then centrifuged at 15 000*g* for 30 minutes at 2°C. The cell pellet was solubilized with the appropriate buffers for sodium dodecyl sulfate‐polyacrylamide gel electrophoresis (SDS‐PAGE) and starch degrading activity. The protein in the supernatant was precipitated by adding 1 vol of acetone (−20°C), 4 vol of methanol, and 1 vol of chloroform. Then, 3 vol of water was added for phase separation. After centrifugation at 15 000*g* for 10 minutes at 2°C, the upper phase was removed, and 3 vol of methanol were added. After centrifugation at 15 000*g* for 10 minutes at 2°C, the upper phase was removed again, and the solid sediment was air‐dried.^15^, ^16^ The extract from the supernatant was also solubilized with the appropriate buffers for SDS‐PAGE and starch degrading activity. The proteins in the culture medium before and after cell culture were precipitated by the same method.

### 2.4 Western blot analysis

Cell pellet and extract and culture medium samples prepared as described above were suspended in TBS. Protein concentrations in the samples were determined using a Pierce BCA protein assay kit with bovine serum albumin as a standard. After boiling at 95°C for 5 minutes with sample buffer ×5 for SDS‐PAGE, the aliquots were loaded onto a 9.5% polyacrylamide gel for SDS‐PAGE.^17^ One gel was transferred to a polyvinylidene fluoride membrane and immunostained with rabbit anti‐HPA IgGs (diluted to 1:1000) and then HRP‐conjugated goat anti‐rabbit IgGs (diluted to 1:500). The membrane was subsequently reacted with 3,3′diaminobenzidine tetrahydrochloride (0.2 mg/mL).^3^ The other gel was stained for proteins using Sypro Ruby.

### 2.5 Starch degrading activity

Starch degrading activity as an assay for α‐amylase enzyme activity was measured by adjusting the method of Bernfeld to a small scale as described previously.^2^ The cell pellet and extract were suspended in 20 mM phosphate buffer, pH 6.9. Protein concentrations in the cell pellet and extract were determined using a Pierce BCA protein assay kit with bovine serum albumin as a standard. The cell lysate sample as an enzyme and 1% soluble starch in 20 mM phosphate buffer saline, pH 6.9, as a substrate were prewarmed separately to 37°C for 15 minutes. The cell sample (1 vol) and 1% soluble starch (2 vol) were mixed and incubated at 37°C for 30 minutes. Ice‐cold dinitrosalicylic acid reagent (2 vol) was added to stop the reaction of the enzyme in the cell samples. The dinitrosalicylic acid reagent was prepared by sequentially adding potassium sodium (+)‐tartrate (15 g) and then 2 N NaOH (10 mL) to 3,5‐dinitrosalicylic acid (20 mg/mL). Water was added to the reagent up to 50 mL. The reaction mixture (50 μL) was diluted with water (200 μL) in a 96‐well plate (Iwaki, Shizuoka, Japan), and then measured at 540 nm using a microplate reader (Vient, DS Pharma Biomedical, Osaka, Japan). The control was used samples had been treated at 98°C for 30 minutes, before the activity assay. Maltose was used as a standard for reducing sugar.

### 2.6 Real‐time polymerase chain reaction

Caco‐2 cells were cultured in 24‐well plates for 0 to 21 days and washed twice with PBS and homogenized in TRIzol. After total RNA was extracted from the TRIzol mixture according to the manufacturer's instructions, the concentration was measured using a Nanodrop spectrophotometer (Thermo Fisher Scientific). cDNAs were synthesized with random primers, dNTPs mixture, and ReverTra Ace, using a Takara PCR Thermal Cycler Dice. cDNA synthesis comprised annealing at 30°C for 10 minutes, transcription at 42°C for 60 minutes, and denaturing at 100°C for 5 minutes and then maintenance at 4°C.

Then, quantitative real‐time PCR was performed using a 7300 or 7500 Real‐Time PCR System (Applied Biosystems, Thermo Fisher Scientific). The reaction mixture, which consisted of 5 µL of the cDNA solution in Tris‐EDTA (Qiagen, Hilden, Germany), 6.25 µL of Power SYBR Green PCR Master Mix, 0.2 µL each of 50 µM forward primer and reverse primer, was added to a MicroAmp Optical 96‐well Reaction Plate (Watson Co, Ltd, Hyogo, Japan). Forward and reverse primers for the gene of α‐amylase and differentiation markers (SI, dipeptidylpeptidase‐4 (DPP‐4), E‐cadherin, and villin) were based on previous reports^18^, ^19^, ^20^ and prepared by Eurofines Genomics (Tokyo, Japan) (Table 1). Data were normalized by using glyceraldehyde‐3‐phosphate dehydrogenase (GAPDH) as an internal control.^21^

**Table 1:** Sequences of primers for real‐time PCR

Genes	Sequence	Reference
GAPDH	Forward: 5′‐TGAAGGTCGGAGTCAACGGAT‐3′	Sachs‐Barrable et al^21^
	Reverse: 5′‐TCGCTCCTGGAAGATGGTGAT‐3′	
α‐Amylase	Forward: 5′‐GTGGAAGTTACTTCAACCCTGGAA‐3′	Kang et al^19^
	Reverse: 5′‐ACATTTACCATCATTAAAATCCCATCCA‐3′	
SI	Forward: 5′‐CATCCTACCATGTCAAGAGCCAG‐3′	Cheng et al^20^
	Reverse: 5′‐GCTTGTTAAGGTGGTCTGGTTTAAATT‐3′	
DPP‐4	Forward: 5′‐GGCGTGTTCAAGTGTGGAAT‐3′	Adnan et al^18^
	Reverse: 5′‐TCTTCTGGAGTTGGGAGACC‐3′	
E‐cadherin	Forward: 5′‐TGATCGGTTACCGTGATCAAAA‐3′	Adnan et al^18^
	Reverse: 5′‐GTCATCCAACGGGAATGCA‐3′	
Villin	Forward: 5′‐AGCCAGATCACTGCTGAGGT‐3′	Adnan et al^18^
	Reverse: 5′‐TGGACAGGTGTTCCTCCTTC‐3′	

Abbreviations: DPP‐4, dipeptidylpeptidase‐4; GAPDH, glyceraldehyde‐3‐phosphate dehydrogenase; SI, sucrase‐isomaltase.

### 2.7 α‐Amylase activity

α‐Amylase‐specific activity in the cell extract and culture medium were measured using an α‐amylase assay kit, which can specifically measure the activity of α‐amylase without the influence of glucoamylase or α‐glucosidase in the sample. Caco‐2 cells were seeded at 5.0 × 10^4^ cells/cm^2^ in six‐well plates. The medium was changed every 2 to 3 days, and the medium was last changed 48 hours before collection. After culture for 7 to 21 days, the medium was collected, and the cells were washed with PBS twice and scraped into cold PBS. The cells in each well were homogenized using a BioMasher III (Nippi Inc, Tokyo, Japan) in 100 μL TBS containing with 1 mM CaCl_2_ on ice and centrifuged at 6000*g* at 4°C for 1 minute. Two milliliters of the culture medium from each well was concentrated to 100 μL and buffered with TBS containing with 1 mM CaCl_2_ using an AmiconUltra‐0.5ml‐10K (Merck Millipore Ltd, County Cork, Ireland). The substrate solution including N3‐G5‐β‐2chloro4nitro phenol (CNP) and the enzyme solution including both glucoamylase and α‐glycosidase were mixed at the volume ratio of 1:1 and preincubated at 37°C for 5 minutes. Four microliters of the cell or medium sample was added to 40 μL of the mixture and incubated at 37°C for 30 minutes. After adding 80 μL of the stop solution including sodium carbonate, absorbance was measured at 400 nm. Pig pancreatic α‐amylase solution (0.3‐10 nM) was used as a standard. Protein concentrations in the samples were determined using a Pierce BCA protein assay kit with bovine serum albumin as a standard. Amylase concentrations (pmol/mg protein) were calculated from the absorbance at 400 nm with the α‐amylase standard and protein concentration of each sample.

### 2.8 Immunofluorescence staining

Caco‐2 cells seeded at 5 x 10^4^ cells/cm^2^ were cultured for 7 or 28 days. The cells were permeabilized after fixation and then stained with anti‐HPA IgGs as the first antibody and Alexa Fluor 488‐conjugated goat antirabbit IgG (H+L) as the second antibody as described previously.^3^ The staining was observed using an LSM710 confocal scanning microcope (Carl Zeiss, Inc., Oberkochen, Germany).^3^

### 2.9 Reverse transcription‐PCR and digestion with restriction enzymes

Total RNA was extracted from Caco‐2 cells that had been seeded at 5 × 10^4^ cells/cm^2^ and cultured for 21 days, mixed with PrimeScript® One Step RT‐PCR Kit (Takara Bio Inc, Shiga, Japan), and amplified using a Takara PCR Thermal Cycler Dice. PCR primers used were: #1 primer for the *Pst*I site, #2 primer for the *Hae*II site, and #3 primer for the *Bam*HI site. These three sets of primers had the same sequences as previously reported.^22^ Total RNA was reverse‐transcribed at 50°C for 30 minutes, and then denatured at 94°C for 2 minutes. Amplification was repeated for 40 cycles, which consisted of denaturing at 94°C for 30 seconds, annealing at 55°C for 30 seconds, and extension at 72°C for 30 seconds. After that, DNA was extracted with phenol/chloroform and precipitated by ethanol. Purified DNA was treated with *Pst*I, *Hae*II, or *Bam*HI at 37°C for 1 hour. Then, restriction products were analyzed by 1.2% agarose gel electrophoresis.

### 2.10 RNAi

Caco‐2 cells were transfected with amylase siRNA using siLentFect as described previously.^23^, ^24^, ^25^ Amylase siRNA (h) is a pool of 19 to 25 nt siRNAs designed to specifically knock down three gene expressions of human *AMY1A*, *AMY2A*, and *AMY2B*. siRNA, amylase siRNA (h) or control siRNA‐A, was diluted to 2 μM with Opti‐MEM and preincubated for 5 minutes at room temperature. siLentFect was diluted at the volume ratio of 4:21 with Opti‐MEM and preincubated for 5 minutes room temperature. After the siRNA and siLentFect preincubated separately had been mixed in equal volumes and incubated at room temperature for 30 minutes, 10 or 50 μL of the mixture was dispensed onto 96‐ or 24‐well plates respectively as the transfection reagent. In addition, Caco‐2 cells lifted from dishes using trypsin‐EDTA were washed with Opti‐MEM and resuspended in Opti‐MEM. The cell suspension (90 or 450 μL) was added to 96‐ or 24‐well plates, respectively, and mixed with the transfection reagent in the wells to 0.5‐2 × 10^5^ cells/cm^2^. Mock cells went through the transfection process without the addition of siRNA. These cells were cultured for 3 days in a humidified 37°C incubator with 5% CO_2_ atmosphere.

### 2.11 MTT assay

The culture medium in the 96‐well plates was changed to 100 μL of Opti‐MEM, and 10 μL of 5 mg/mL MTT solution was added to well. After incubation at 37°C for 2.5 hours, the cells on the wells were observed under a light microscope at ×10 magnification. The MTT solution was removed, and 100 μL of MTT solvent (40 mM HCl, 0.1% NP40 in isopropanol) was added. The plate was wrapped in foil and shaken to fully dissolve the MTT formazan. The absorbance of the formazan solution in each well was measured at 590 nm. As a standard, the cells were seeded at 0.03‐2 × 10^5^ cells/cm^2^ in a 96‐well plate and cultured under the same conditions.

### 2.12 Statistical analysis

All cell experiments were performed independently at least twice. The results are expressed as means ± standard error (SE). For statistical analyses of data, unpaired *t‐*test or one‐way analysis of variance with post‐hoc test was used as appropriate. All analyses were performed using IBM SPSS Statistics software for Windows, Version 23.0. (IBM Corp, Armonk, NY). Differences of *P*< 0.05 were considered statistically significant.

## 3 RESULTS

### 3.1 α‐Amylase is expressed at the highest level in duodenum after pancreas in normal human tissues

The expression of α‐amylase in normal human tissues was quantified by real‐time PCR using Human MTC Panels I and II and a Digestive System MTC Panel in first‐strand cDNA preparations from RNA from the tissues.

**Figure 1:**
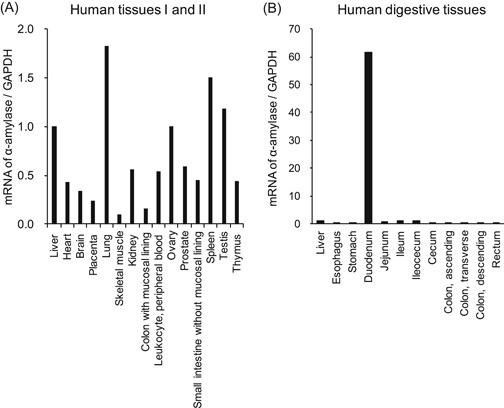
Expression of α‐amylase in normal human tissues. The mRNA expressions of α‐amylase in human normal tissues were quantified by real‐time PCR. The α‐amylase/GAPDH ratio for liver was set as the baseline value to which all transcript levels were normalized. mRNA of α‐amylase/GAPDH in human tissues I and II across Human MTC Panel I and II (A) and in human digestive tissues across the Human Digestive System MTC Panel (B). GAPDH, glyceraldehyde‐3‐phosphate dehydrogenase; mRNA, messenger RNA; MTC, multiple tissue cDNA; PCR, Polymerase chain reaction

The mRNA expression level of α‐amylase in the pancreas was 1063 times higher than that of the liver (data not shown). Therefore, figures show mRNA expression of α‐amylase in tissues excluding the pancreas in a ratio relative to the liver. All mRNA levels were below 2.0, and the levels of 1.0 or more were lung, ovary, spleen, and testis in Panels I and II (Figure 1A). On the other hand, the expression level of α‐amylase was remarkably high in the duodenum in the Digestive System Panel, 61.8 times higher than that in the liver (Figure 1B). Expression levels in all other digestion tissues were all below 1.0. These results show that after the pancreas, the tissue with the highest α‐amylase expression was the duodenum.

### 3.2 α‐Amylase is expressed in Caco‐2 cells

**Figure 2:**
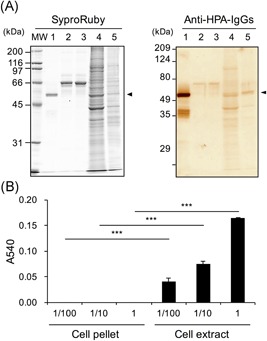
Western‐blotting for α‐amylase and starch‐degrading activity in Caco‐2 cells and culture medium. Caco‐2 cells were seeded at 5 × 10^4^ cells/cm^2^ in dishes (150 × 20 mm) and cultured for 6 days. The cells and culture medium before and after culture (2  mL each) were prepared as described in Section 2. A, Western‐blotting for α‐amylase: The samples were boiled with SDS‐PAGE sample buffer including 2‐mercaptoethanol, and 10 μL aliquots were used for Western blot analysis as described in Section 2. Left, Protein staining using Sypro Ruby. Right, Immunostaining for α‐amylase using rabbit anti‐HPA IgGs and HRP‐conjugated goat anti‐rabbit IgGs, MW: molecular weight maker, Lane 1, purified pig pancreatic α‐amylase (0.8 μg/lane); 2, medium before culture (1.7  μg protein/lane); 3, medium after culture (1.8 μg protein/lane); 4, cell pellet (12 μg protein/lane); 5, cell extract (2.3 μg protein/lane). B, Starch‐degrading activity: Caco‐2 cells were cultured in dishes (150 × 20 mm) for 6 days. The cell pellet and extract were separately solubilized in 500 μL of 20 mM phosphate buffer, pH 6.9. The protein concentrations of the cell pellet and extract were 0.227 and 1.16 mg/mL, respectively. The cell pellet and extract (1, 10, and 100 times dilution) were used as enzyme samples (1 vol was 15 μL) as described in Section 2. HPA, human pancreatic α‐amylase; IgG, immunoglobulin G; SDS‐PAGE, sodium dodecyl sulfate‐polyacrylamide gel electrophoresis ***P < 0.001 vs cell pellet by unpaired *t* test

We used Caco‐2 cells as a human intestinal epithelial cell to demonstrate the expression of α‐amylase in the intestine and examine its role. The α‐amylase expression in the cells was first demonstrated by Western blot analysis of the culture medium, cell pellet, and cell extract. The cell extract contained all components except for those of the cell pellet, which was rich in nuclei and cell debris.^26^ As shown in Figure 2A, many protein bands in both the cell pellet and extract were stained using Sypro Ruby, and the 54 kDa band, which was reactive with anti‐HPA IgGs, corresponding to the molecular weight of purified α‐amylase (lanes 1, 4, and 5, indicated by arrows in Figure 2A). Bands at about 45 kDa, immunostained by anti‐HPA IgGs, are degradation fragments of α‐amylase (lanes 1, 4, and 5 in Figure 2A). No band was stained in the medium either before or after culture by anti‐HPA IgGs at 54 kDa or about 45 kDa (lanes 2 and 3 in Figure 2A). To determine whether the α‐amylase detected by Western blot analysis has enzyme activity or not, starch‐degrading activity was examined. As shown in Figure 2B, the enzyme activity detected in the cell extract was concentration‐dependent, while the activity was not detected in the cell pellet at any concentration. These results show that active α‐amylase is expressed in Caco‐2 cells and detected in the cell extract but not the medium.

### 3.3 Expression of α‐amylase increases with differentiation to small intestinal epithelial cells

As shown in Figure 2, the expression of α‐amylase was detected in Caco‐2 cells cultured for 6 days. Although Caco‐2 was originally a human colon epithelial cell line, the cells exhibit characteristics of small intestinal epithelial cells with a brush‐border layer when they are cultured for 14 to 21 days after confluence. Therefore, differentiated Caco‐2 cells are commonly used as a for human small intestinal epithelial assays in vitro.^25^, ^27^ We examined the effects of differentiation on the α‐amylase expression in Caco‐2 cells.

**Figure 3:**
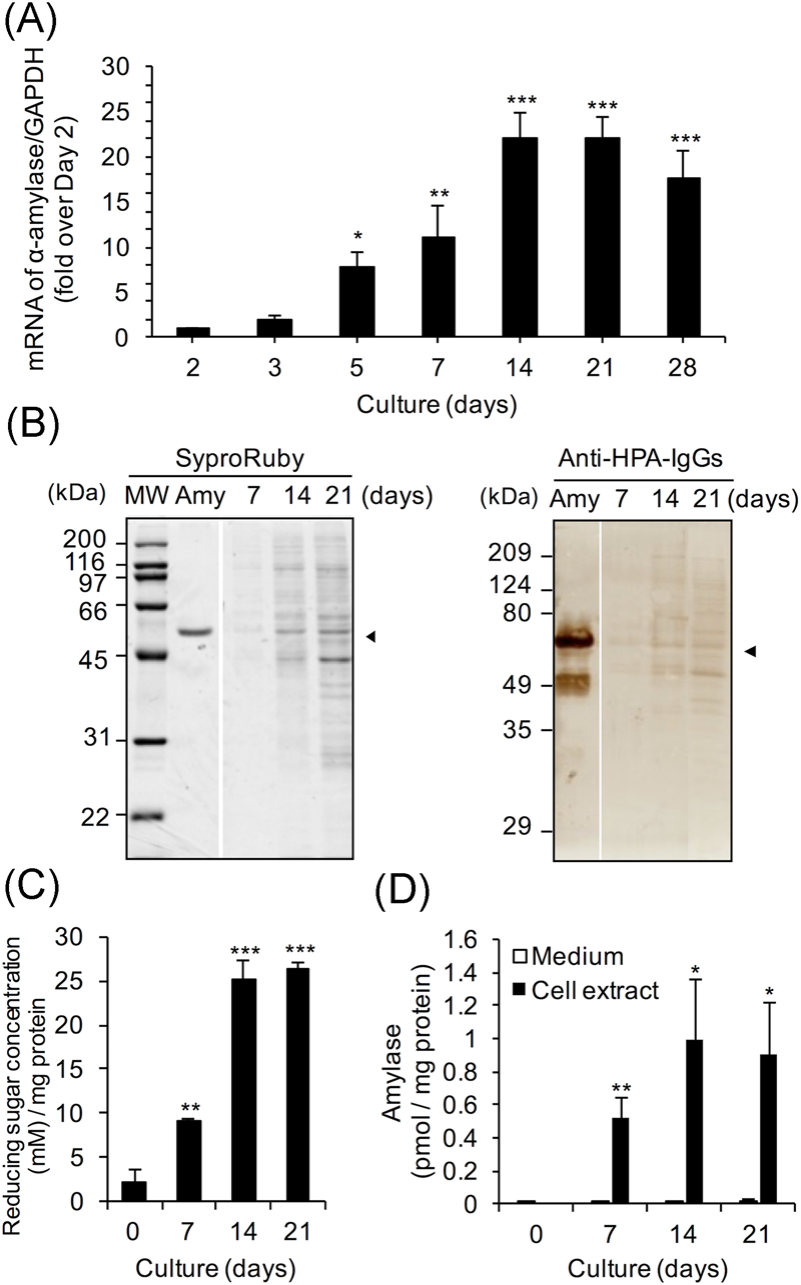
Expression of α‐amylase in Caco‐2 cells cultured 28 days. The cells were seeded at 5 × 10^4^ cells/cm^2^. The first medium change was made 2 days after seeding, and then the medium was changed every 2 to 3 days until 21 or 28 days. A, mRNA expression of α‐amylase: The cells were seeded and cultured in six‐well plates. The expression of α‐amylase was determined by real‐time PCR. The expression for days 3 to 28 (ΔΔ*C*_t_ method vs that cultured for day 2) is shown; mean ± SE for 4 to 5 independent experiments. *P  < 0.05, **P  < 0.01, ***P < 0.001 vs day 2 by one‐way ANOVA with Dunnett's post‐hoc test. B, Western blot analysis for α‐amylase: The cells were seeded and cultured in a dish (100 × 20  mm). The cell extracts were boiled with SDS‐PAGE sample buffer ×5 including 2‐mercaptoethanol, and aliquots (10 μL each) were used for Western blot analysis as described in Section 2, left. Protein staining using Sypro Ruby, right. Immunostaining for α‐amylase using rabbit anti‐HPA IgGs and HRP‐conjugated goat anti‐rabbit IgGs. MW denotes molecular weight markers, Amy is purified pig pancreatic α‐amylase (0.8 μg/lane), 7 to 21 days: Extract of cells cultured for 7, 14, or 21 days (1.4, 2.4, and 3.7  μg protein/lane each). C–D, Enzymatic activity of α‐amylase: The cells were seeded and cultured in 24‐well plates for 7 to 21 days. The cell extracts were assayed for (C) starch degrading activity (1 vol was 10 μL) and (D) α‐amylase activity as described in Section 2. The activity for days 7 to 21 is shown as mean ± SE for three independent experiments. ANOVA, analysis of variance; HPA, human pancreatic α‐amylase; HRP, horseradish peroxidase; IgG, immunoglobulin G; mRNA, messenger RNA; MW, molecular weight; SDS‐PAGE, sodium dodecyl sulfate‐polyacrylamide gel electrophoresis; □, medium; ■, cell extract. (C) **P < 0.01, ***P  <  0.001 vs day 0 by one‐way ANOVA with Dunnett's post‐hoc test. (D) *P < 0.05, **P < 0.01 vs medium by paired *t* test

mRNA levels of α‐amylase in the cells increased depending on the number of culture days until 14 days (Figure 3A). The highest levels of amylase mRNA in cultures were found at 14 and 21 days, and the mRNA levels at days 14 and 21 were 22.1‐ and 21.9‐fold, respectively, those at day 2. The expression on day 28 was also significantly higher than on day 2, although a tendency was observed to decrease as compared to days 14 and 21. The staining intensities of 54 kDa bands corresponding to α‐amylase also increased depending on the number of culture days from the detection of protein levels by Western blot analysis using anti‐HPA IgGs (Figure 3B, indicated with black arrowheads).

The starch degrading activity of α‐amylase in the cell extract increased culture day‐dependently. The activity was markedly increased at 14 days, and the high activity was maintained at 21 days (Figure 3C). Additionally, the α‐amylase‐specific activity was detected mainly in the cell extract, while the culture medium had little α‐amylase activity (Figure 3D). In the cell extract, the activity at day 14 was the highest, and there is no activity at day 0 which was before seeding the cells.

**Figure 4:**
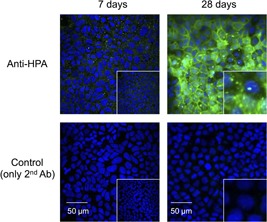
Immunostaining for α‐amylase in Caco‐2 cells. Caco‐2 cells were seeded at 5 × 10^4^ cells/cm^2^ and cultured on microcover glasses in 24‐well plates for 7 or 28 days. The cells were permeabilized after fixation and incubated with rabbit anti‐HPA IgGs (diluted to 1:150) and visualized by Alexa Flour488‐conjugated goat anti‐rabbit IgGs (diluted to 1:200, green fluorescence). Nuclei were counterstained with DAPI. Controls were incubated with only the secondary antibody without the anti‐HPA IgGs. The lower right magnification is ×6.67. Scale bars = 50 μm. DAPI, 4′,6‐diamidino‐2‐phenylindole; HPA, human pancreatic α‐amylase; IgG, immunoglobulin G

Further, the location of α‐amylase in the cells was observed by confocal microscopy. α‐Amylase was stained as intracellular puncta after culturing for 7 days (Figure 4). Its staining after culture for 28 days was remarkably stronger than after culture for 7 days. There was no staining for α‐amylase using only the second antibody as a control.

These results clearly show that α‐amylase is expressed differentiation‐dependently at both the mRNA and protein levels in Caco‐2 cells.

### 3.4 α‐Amylase isozyme is identified as *AMY2B*

**Figure 5:**
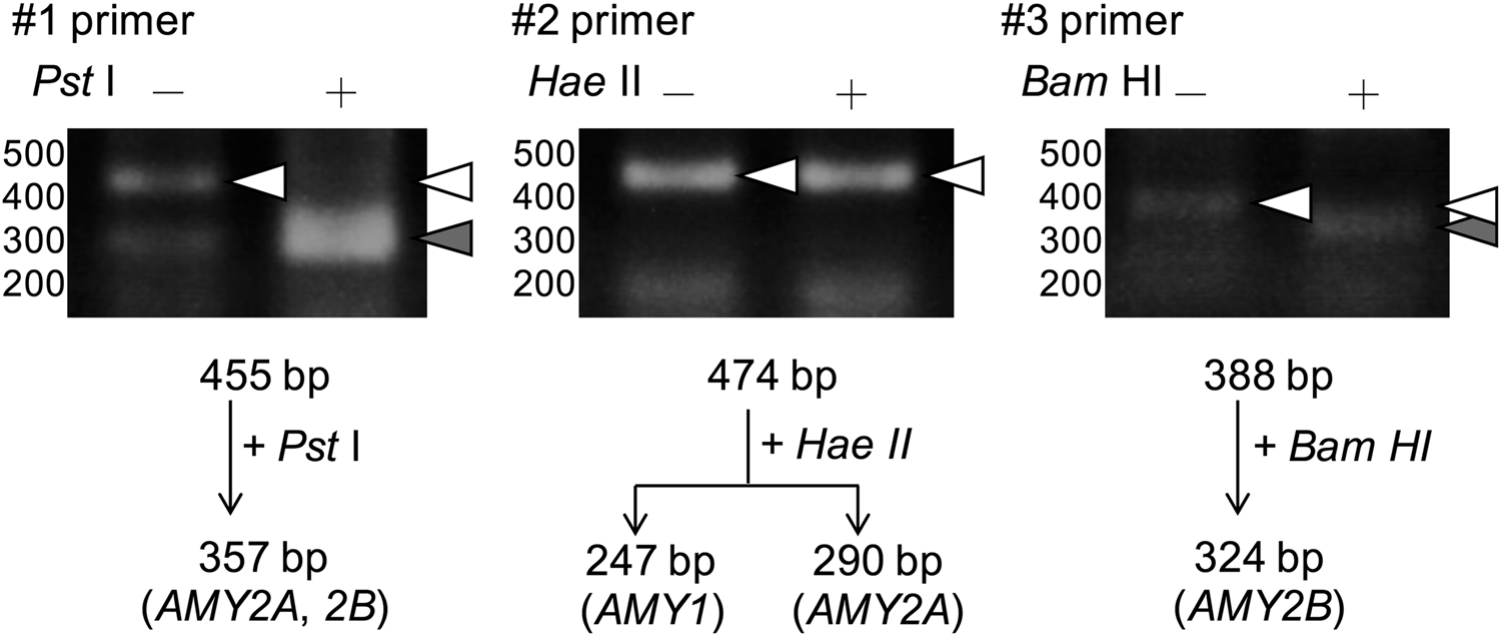
Identification of α‐amylase isozyme expressed in differentiated Caco‐2 cells. Caco‐2 cells were seeded at 5 × 10^4^ cells/cm^2^ and cultured in six‐well plates for 21 days. Total RNAs from cultured Caco‐2 cells were separately reacted with three sets of primers (#1, #2, #3) in RT‐PCR as described in Section 2. The RT‐PCR products were treated without (−) or with (+) *Pst*I, *Hae*II, or *Bam*HI. Arrowheads indicate the positions of migrated fragments that were cleaved (filled triangle) or not cleaved (open triangle), respectively. RT‐PCR, reverse transcription polymerase chain reaction

There are two major isozymes of α‐amylase, pancreatic α‐amylase (HPA) and human salivary α‐amylase (HSA). The salivary α‐amylase is encoded by *AMY1*, and pancreatic α‐amylases are encoded by *AMY2A* or *AMY2B*.^11^ To identify the α‐amylase isozymes expressed in Caco‐2 cells, we performed a restriction endonuclease assay.^22^ As shown in Figure 5, it was ascertained that the α‐amylase is encoded by *AMY2A* or *AMY2B* if the 455 bp fragment amplified by #1 primers is restricted to 357 bp by *PstI*. In the same way, the α‐amylase is encoded by *AMY1* or *AMY2A* if the 474 bp fragment amplified by #2 primers is restricted to 247 bp or 290 bp by *Hae II*. The α‐amylase is encoded by *AMY2B* if the 388 bp fragment amplified by #3 primers is restricted to 324 bp by *Bam*HI.

A DNA fragment amplified by RT‐PCR with #1 primers was cleaved to 357 bp by *Pst* I, indicating that the α‐amylase expressed in Caco‐2 was either *AMY2A* or *AMY2B* (Figure 5, left). DNA fragments amplified by RT‐PCR with #2 primers were not cleaved by *HaeII*, indicating that the α‐amylase expressed was neither *AMY1* nor *AMY2A* (Figure 5, middle). DNA fragments amplified by RT‐PCR with #3 primers that were not cleaved by *Bam*HI were cleaved to 324 bp, supporting the hypothesis that the α‐amylase expressed is *AMY2B* (Figure 7, right). These results demonstrated the α‐amylase expressed in Caco‐2 is encoded by *AMY2B*.

### 3.5 Suppression of α‐amylase expression by siRNA inhibits cell differentiation and proliferation

To determine the biological significance of α‐amylase expression in Caco‐2, we tried to suppress the expression of α‐amylase by RNAi. The above results show that α‐amylase expression increases as cells differentiate to small intestine cells. Therefore, we hypothesized that the α‐amylase expressed in Caco‐2 cells may contribute to the differentiation to small intestine cells, and measured the mRNA levels of four kinds of differentiation markers of the small intestine. SI and dipeptidylpeptidase‐4 (DPP‐4) are differentiation marker proteins of intestinal epithelial cells, and both are expressed in the brush border of the intestinal tract,^28^ while E‐cadherin is a component in intestinal epithelium cells that mainly contain adherens junctions,^29^ and villin is an actin‐binding protein localized in intestinal brush borders.^30^

**Figure 6:**
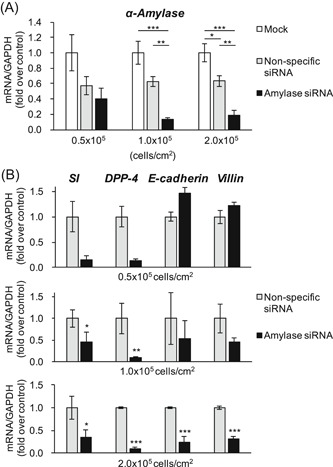
Effect of α‐amylase suppression on cell differentiation. Caco‐2 cells (0.5‐2.0 × 10^5^ cells/cm^2^) were transfected with none as a mock, nonspecific siRNA as a control, or the siRNA targeting α‐amylase. A, mRNA expression of α‐amylase. *P < 0.05, **P < 0.01, ***P < 0.001, one‐way ANOVA with Tukey's post‐hoc test. B, mRNA expression of differentiation makers (SI, DPP‐4, E‐cadherin, and villin). These expressions (ΔΔ*C*_t_ method vs mock) are shown as mean ± SE for three independent experiments. *P < 0.05, **P < 0.01, ***P < 0.001 vs nonspecific siRNA by paired *t*‐test. ANOVA, analysis of variance; DPP‐4, dipeptidylpeptidase‐4; mRNA, messenger RNA; SI, sucrase‐isomaltase; siRNA, small interfering RNA

The expression of α‐amylase was suppressed in cell seedings at both 1.0 × 10^5^ and 2.0 × 10^5^ cells/cm^2^, while the expression in the cell seeding at 0.5 × 10^5^ cells/cm^2^ was not suppressed sufficiently (Figure 6A). In the cell seeding at 0.5 × 10^5^ cells/cm^2^, there was no change in the expression of not only α‐amylase but also all of those differentiation markers, especially E‐cadherin and villin (Figure 6B). The expressions of SI and DPP‐4 were decreased by the suppression of α‐amylase expression at 1.0 × 10^5^ cells/cm^2^, but those of E‐cadherin and villin were not. In the cell seeding at 2.0 × 10^5^ cells/cm^2^, the expressions of all differentiation makers were clearly inhibited by the suppression of α‐amylase expression. These results show that the suppression of α‐amylase expression inhibits the expression of differentiation markers, suggesting that the α‐amylase expressed in the cell is necessary to induce cell differentiation.

**Figure 7:**
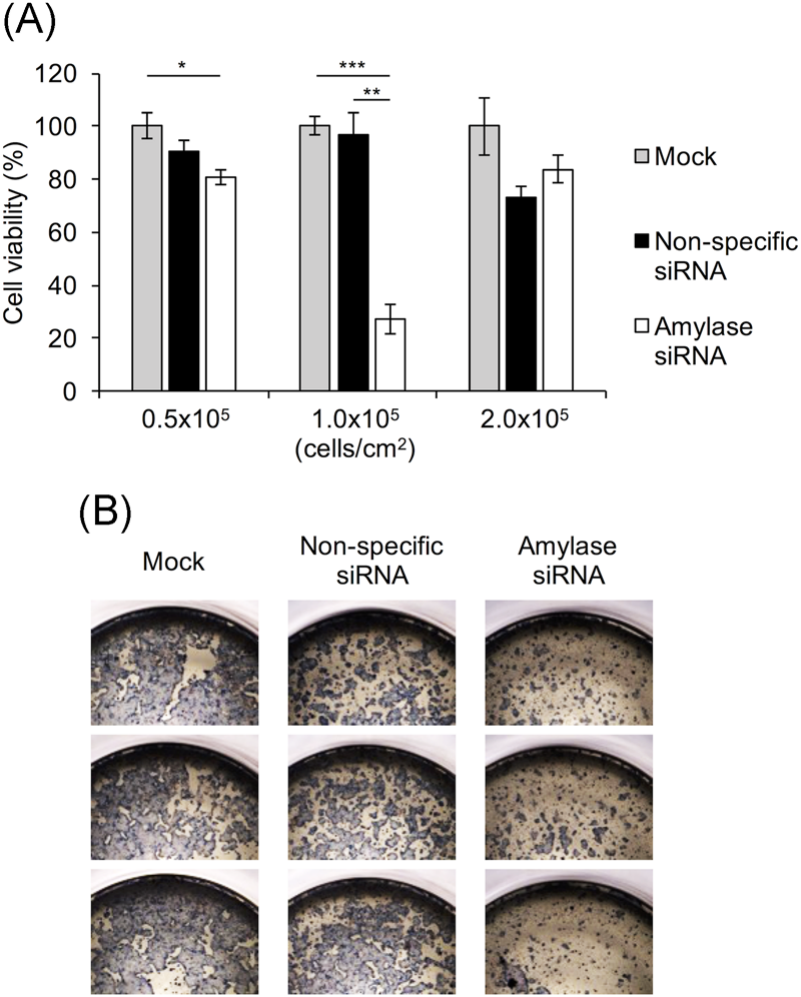
Effect of α‐amylase suppression on cell proliferation. Caco‐2 cells (0.5‐2.0 ×  10^5^ cells/cm^2^) were transfected with none as a mock, nonspecific siRNA as a control, or the siRNA targeting α‐amylase. A, Cell viability measured by MTT assay. The cell viability (% of mock) is shown as mean ± SE for three independent experiments. *P  < 0.05, **P  < 0.01, ***P  < 0.001 by one‐way ANOVA with Tukey's post‐hoc test. B, The cells in the 96‐well plates stained with MTT after the cells (1.0 × 10^5^ cells/cm^2^) were transfected (*n* = 3). ANOVA, analysis of variance; MTT, thiazolyl blue tetrazolium bromide; SE, standard error; siRNA, small interfering RNA

In the process of examining the influence of cell differentiation due to the suppression of α‐amylase expression, we noticed that not only cell differentiation but also cell proliferation was observed by suppressing the expression of α‐amylase. Therefore, to clarify the effects of the suppression of α‐amylase expression on cell proliferation, an MTT assay was performed. The suppression of α‐amylase expression significantly inhibited cell proliferation in the cell seeding at 1.0 × 10^5^ cells/cm^2^ (Figures 6A, 7A, and 7B). These results show that suppression of α‐amylase expression also inhibits cell proliferation.

These results suggest that intracellularly expressed α‐amylase is essential for both cell differentiation to small intestinal epithelial cells and cell proliferation.

## 4 DISCUSSION

Here, we examined whether α‐amylase is expressed in normal human tissues and the intestinal epithelial cell Caco‐2. α‐Amylase was found to be expressed at the highest mRNA level in the duodenum in human normal tissues after the pancreas (Figure 1). We also found that active α‐amylase is expressed in Caco‐2 cells and that the expression increased depending on the culture durations of the cells by using real‐time PCR, Western blot analysis, enzyme activity assays, and confocal microscopic images for α‐amylase (Figures 2, 3, 4). These results suggest that α‐amylase is expressed in epithelial cells of the small intestine rather than the colon because Caco‐2 cells differentiate in long‐term culture to exhibit characteristics of small intestinal epithelial cells, while they originally had characteristics of colon epithelial cells.^4^ This suggestion is demonstrated by the result that mRNA expression of α‐amylase in the duodenum was significantly higher in normal human digestive tissues and 140 to 340 times higher than in the colon (Figure 1).

In almost all the studies from the 1980s to the 2000s,^8^, ^9^, ^10^, ^14^ α‐amylase activity was measured by using Blue Starch as the substrate^31^ or by wheat‐germ inhibition, which could not detect the activity of α‐amylase without detecting the contamination of glucoamylase or α‐glucosidase in samples such as cells and tissues. Therefore, we used two kinds of assays: starch‐degrading activity by the Bernfeld method on a small scale^1^, ^2^ and an α‐amylase assay kit, which can measure α‐amylase‐specific activity without the interference of glucoamylase or α‐glucosidase in the sample. The α‐amylase activity was detected in cell extracts but not in the culture medium (Figures 2, 3, and 3D), suggesting that the active α‐amylase is expressed mainly in the cells but not secreted out of the cell.

A restriction endonuclease assay also showed that the α‐amylase expressed in Caco‐2 cells was identified as *AMY2B* (Figure 5). *AMY2B* is one of the pancreatic α‐amylases and is expressed at a low level in normal human liver.^8^ The biological significance of these α‐amylase expressions in the tissues including the liver is still unknown, while expression of the pancreas and saliva α‐amylases is responsible for converting polysaccharides such as starch.^32^ It has been suggested that the α‐amylase expressed in the liver might contribute to glycogen metabolism because amylase activity accelerates during periods of hypoglycemia such as fasting and during liver regeneration after partial hepatectomy.^33^ It is reported that hepatic expression of the α‐amylase gene may be a biomarker of an early stage of obesity because α‐amylase levels in the liver were increased in obese mice.^34^

With this background, we found in this study that suppression of the expression of α‐amylase reduces both the expressions of several differentiation marker proteins and cell viability (Figures 6 and 7). These results suggest that the expression of α‐amylase is essential for cell proliferation and differentiation. The expression of α‐amylase was suppressed by seeding at 1 × 10^5^ and 2 × 10^5^ cells/cm^2^, but inhibition of the expressions of differentiation marker proteins was detected at 2 ×  10^5^ cells/cm^2^ but not at 1 × 10^5^ cells/cm^2^. As an interpretation of the results, it is considered that the cells are hardly differentiated at 1 × 10^5^ cells/cm^2^ because there are not enough cells for differentiation, while the cells reached a level of confluence adequate for cell differentiation at 2 × 10^5^ cells/cm^2^. On the other hand, inhibition of cell viability was observed at 1 × 10^5^ cells/cm^2^ but not at 2 × 10^5^ cells/cm^2^. The reason may be that there are too many cells to detect the difference because the cell viability of the standard reached a plateau at the high seeding density.

Intestinal epithelial cells have the shortest life span among all the cells comprising the body. They have the most rapid turnover of all tissues in the body, and their regulation is considered to be very important to keep the intestinal epithelium healthy.^35^, ^36^ The homeostasis is maintained by replacement of differentiated intestinal epithelial cells continuously and rapidly, and its replacement was supported by replication of undifferentiated cells and subsequent differentiation.^37^ Our results indicate that α‐amylase could induce proliferation and differentiation of small intestine epithelial cells.

**Figure 8:**
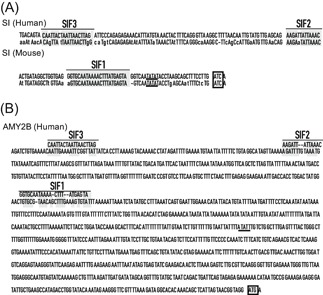
Similar SIF sequences within human *AMY2B* promoter. A, SIFs (SIF1‐3) within the SI promoter are shown as previously reported.^38^ The human gene is shown above the mouse gene. The bars labeled SIF1‐3 and shaded areas in SIF1‐3 are regions of complete identity between the human and mouse genes. The TATA consensus sequence is underlined. The start codon is indicated by a box. B, Similar sequences of SIFs within the human *AMY2B* promoter. The bars labeled SIF1‐3 and shaded areas in SIF1‐3 are regions of complete identity between the human SIFs and human *AMY2B*. The TATA consensus sequence is underlined. Start codon is indicated by a box. SI, sucrase‐isomaltase; SIF, footprint of SI

This study reports for the first time that α‐amylase is expressed in Caco‐2 cells and that the expression is increased as the cells develop the characteristics of small intestinal epithelial cells. Differentiated Caco‐2 cells have been used as an intestinal epithelium model to study oral absorption and evaluate the mechanisms of nutrient transport.^5^ Our results suggest that α‐amylase might be useful as a marker for the differentiation of Caco‐2 cells into intestinal epithelial cells, similar to SI. The SI promoter has three nuclear protein binding sites, which are SIF1‐3, where SIF indicates a footprint of SI. Each SIF positively regulates transcription of SI in Caco‐2 cells.^38^ We investigated whether or not sequences similar to these three promoters SIF1‐3 were present within the human α‐amylase gene *AMY2B*.^39^ *AMY2B* contains a sequence similar to SIF1‐3, the order of which is SIF3, 2, 1 from the 5′ end, which is the same as the order of the sequence in SI (Figure 8). This suggests that α‐amylase and SI might regulate differentiation of human small intestinal epithelial cells using the same DNA‐binding proteins. We will verify this hypothesis in future research.

In summary, we showed here that α‐amylase is most highly expressed in the duodenum after the pancreas in human normal tissues. The active α‐amylase differentiation‐dependently expressed in Caco‐2 cells was identified as *AMY2B*, and the suppression of α‐amylase expression by siRNA inhibited both cell differentiation and proliferation. From these results, it is expected that these α‐amylase expressions in human epithelial cells may be one of the key molecules for the rapid turnover to maintain a healthy intestinal lumen with homeostasis.

## CONFLICT OF INTERESTS

The authors declare that there are no conflict of interests.

## AUTHOR CONTRIBUTIONS

KD, YT, and KH performed the experiments and analyzed the data. KD and YT wrote the manuscript. KD and TY conceived the study. KD and HO initiated the study. All authors reviewed the manuscript.
